# Crosstalk Between the Unfolded Protein Response, MicroRNAs, and Insulin Signaling Pathways: In Search of Biomarkers for the Diagnosis and Treatment of Type 2 Diabetes

**DOI:** 10.3389/fendo.2018.00210

**Published:** 2018-05-02

**Authors:** Chinar Berry, Megha Lal, B. K. Binukumar

**Affiliations:** ^1^CSIR-Institute of Genomics and Integrative Biology, Delhi, India; ^2^Academy of Scientific and Innovative Research (AcSIR), Delhi, India

**Keywords:** type 2 diabetes, unfolded protein response, endoplasmic reticulum stress, microRNAs, biomarkers

## Abstract

Type 2 diabetes mellitus (T2DM) is a metabolic disorder that is characterized by functional defects in glucose metabolism and insulin secretion. Its complex etiology and multifaceted nature have made it difficult to design effective therapies for early diagnosis and treatment. Several lines of evidence indicate that aberrant activation of the unfolded protein response (UPR) in response to endoplasmic reticulum (ER) stress impairs the β cell’s ability to respond to glucose and promotes apoptosis. Elucidating the molecular mechanisms that govern β cell dysfunction and cell death can help investigators design therapies to halt or prevent the development of T2DM. Early diagnosis of T2DM, however, warrants additionally the identification of potential biomarkers. MicroRNAs (miRNAs) are key regulators of transcriptional processes that modulate various features of insulin signaling, such as insulin sensitivity, glucose tolerance, and insulin secretion. A deeper understanding of how changes in patterns of expression of miRNAs correlate with altered glucose metabolism can enable investigators to develop methods for the early diagnosis and treatment of T2DM. The first part of this review examines how altered expression of specific UPR pathway proteins disrupts ER function and causes β cell dysfunction, while the second part discusses the potential role of miRNAs in the diagnostic and treatment of T2DM.

## Introduction

Diabetes mellitus is a chronic, progressive disorder that results from the body’s inability to either produce sufficient insulin or utilize it efficiently (1). The key feature of the disease is chronic hyperglycemia—chronically elevated blood glucose—which facilitates the development of glucose intolerance and insulin resistance, and impairs the pancreas’ ability to respond to changes in blood glucose concentration (2). Owing to its complex, multifactorial nature, diabetes affects more than just pancreatic function—neuropathy, nephropathy, and retinopathy are common problems that arise as the disease progresses (3, 4).

A rise in the prevalence of diabetes and its associated rate of mortality are an increasing source of concern for health agencies around the world. According to WHO statistics, the global prevalence of diabetes among adults in 2014 was nearly double of what it was in 1980, due, in part, to increasing obesity, sedentary lifestyles, and unhealthy diet (5). The absolute number of people having diabetes nearly quadrupled during this time, so that by 2014, an estimated 422 million people were living with it (5). In 2012, diabetes accounted for the death of 1.5 million people worldwide, with another 2.2 million deaths being the result of complications of high blood glucose (5). Perhaps what is most troubling is that a large proportion of people with diabetes do not even know they have it—India alone had an estimated 70 million diabetics in 2015, of which 36 million went undiagnosed (1).

These staggering figures not only reflect the impact that diabetes has on the global population, but also speak to a dire need for better prevention to reduce this impact. Having effective preventative measures is particularly important in the context of diabetes as 90% diabetics have type 2 diabetes mellitus (T2DM), which, in a large proportion of cases, is preventable (6). T2DM is a metabolic disorder—as opposed to Type 1 diabetes, which has a strong autoimmune, inflammatory component (7)—which develops as a result of either insufficient insulin secretion from pancreatic β cells, or insulin resistance in insulin-sensitive tissues such as liver, adipose tissue, and skeletal muscle (8–10). Already there is evidence to suggest that simple lifestyle changes such as eating healthy, maintaining an appropriate body mass index, and engaging in physical activity can reduce the risk of T2DM (1). And, investigators are currently researching ways to further reduce susceptibility to this disease by altering genetic and epigenetic factors involved in its pathogenesis.

In recent years, endoplasmic reticulum (ER) stress has emerged as a key regulator of transcriptional and translational responses in T2DM. Because proper protein folding is crucial for protein homeostasis and cell survival, the ER maintains an environment that favors it; this process, however, is particularly sensitive and even small changes in the cellular environment can cause proteins to misfold (11, 12). Accumulation of misfolded proteins in the ER disrupts protein homeostasis and causes ER stress. Unresolved ER stress has been implicated in various diseases such as Alzheimer’s disease (13), chronic kidney disease (14), and hepatocellular carcinoma (HCC) (15) because it has a profound impact on cellular function and activates pro-apoptotic pathways inside the cell. An immense demand for insulin makes pancreatic β cells particularly susceptible to ER stress, as a result of which numerous studies have implicated chronic ER stress in β cell dysfunction and cell death in T2DM. The current model for ER stress-mediated development of T2DM proposes that dysregulation of ER stress signaling pathways in β cells inhibits insulin secretion and makes them susceptible to glucotoxicity. By understanding the factors that modulate the interaction between ER stress pathways and insulin signaling, investigators hope to find diagnose to therapeutic targets in T2DM.

The pressing need for early diagnosis of T2DM has also drawn the attention of investigators’ in the field to microRNAs (miRNAs). It has been known for some time that although 90% of the human genome is transcribed, only a small fraction of the transcripts is translated into proteins; the remainder is non-coding RNA (ncRNA) (16). Of the two broad classes of ncRNAs—infrastructural and regulatory—the set of regulatory ncRNAs is crucial for the regulation of gene expression. miRNAs are small, single-stranded ncRNAs that are typically 22 nucleotides in length (17). They regulate gene expression by either cleaving specific mRNAs or repressing their translation. But, they are only one of many types of regulatory ncRNAs; the others include, but are not limited to, small interfering RNAs (siRNAs), Piwi-interacting RNAs, and long non-coding RNAs (17). Because they can modulate over 60% of the protein-coding genes in the human genome, miRNAs have a profound impact on key cellular processes, including differentiation, proliferation, growth, and apoptosis (3, 4). Previous studies have implicated aberrant miRNA regulation in several cardiovascular disease pathologies (18) including hypertension, ischemic heart disease, and heart failure, and various neurological disorders (19), such as Alzheimer’s disease, Parkinson’s disease, and schizophrenia. Several types of cancer—lymphocytic leukemia, HCC, and glioma—are also associated with differential expression of miRNAs (20).

A similar association has emerged between patterns of miRNA expression and T2DM. A wealth of evidence now suggests that levels of miRNAs in insulin-sensitive tissues change with the development of T2DM (21–23). In obese mice, for example, miR-103 and miR-107 are upregulated in the liver and adipose tissue; this upregulation impairs the ability of those tissues to metabolize glucose normally (24). Silencing of both miRNAs, however, restores glucose homeostasis by improving glucose tolerance and insulin sensitivity. In addition to insulin sensitivity, changes in miRNA expression also modulate pancreatic development, islet morphology, and glucose-stimulated insulin secretion (GSIS) in β cells (25). By analyzing trends of miRNA expression in healthy individuals and type 2 diabetics, investigators may be able to use changes that occur at earlier stages of the disease as biomarkers, and design therapies that can prevent the onset of T2DM (25, 26).

## Adaptive and Pro-Apoptotic Responses in Cells under ER Stress

Endoplasmic reticulum stress is the result of accumulation of unfolded or misfolded proteins within the ER (16). It typically occurs when an increased demand for proteins overloads the ER’s protein folding mechanism, which causes unfolded proteins to build up in the lumen. In addition to protein overload, exposure to environmental toxins, viral infections, or mutant proteins also induce ER stress, although it may develop simply because of aging (27). But while several factors influence the development of ER stress, they each trigger the unfolded protein response (UPR)—an adaptive response that works to mitigate ER stress and maintain protein homeostasis.

The UPR can counteract the effects of ER stress on the cell in three distinct ways (27, 28). It firstly upregulates the transcription of genes encoding molecular chaperones such as foldases, which promote protein folding and reduce protein aggregation. Upregulation of the ER-associated degradation (ERAD) pathway additionally contributes to the clearance of unfolded proteins from the ER. But while ER stress signaling increases the expression of cellular factors modulating protein folding and degradation, it attenuates the translation of other cellular proteins to prevent them from aggregating in the ER. Three proteins—inositol-requiring 1 (IRE1), PKR-like Kinase (PERK), and activating transcription factor 6 (ATF6)—mediate the actions of UPR by acting as sensors for the presence of unfolded proteins (2, 27). When activated, each protein initiates a cascade of downstream effects that alters the transcription, translation, and post-translational modification of cellular proteins.

**Figure 1:**
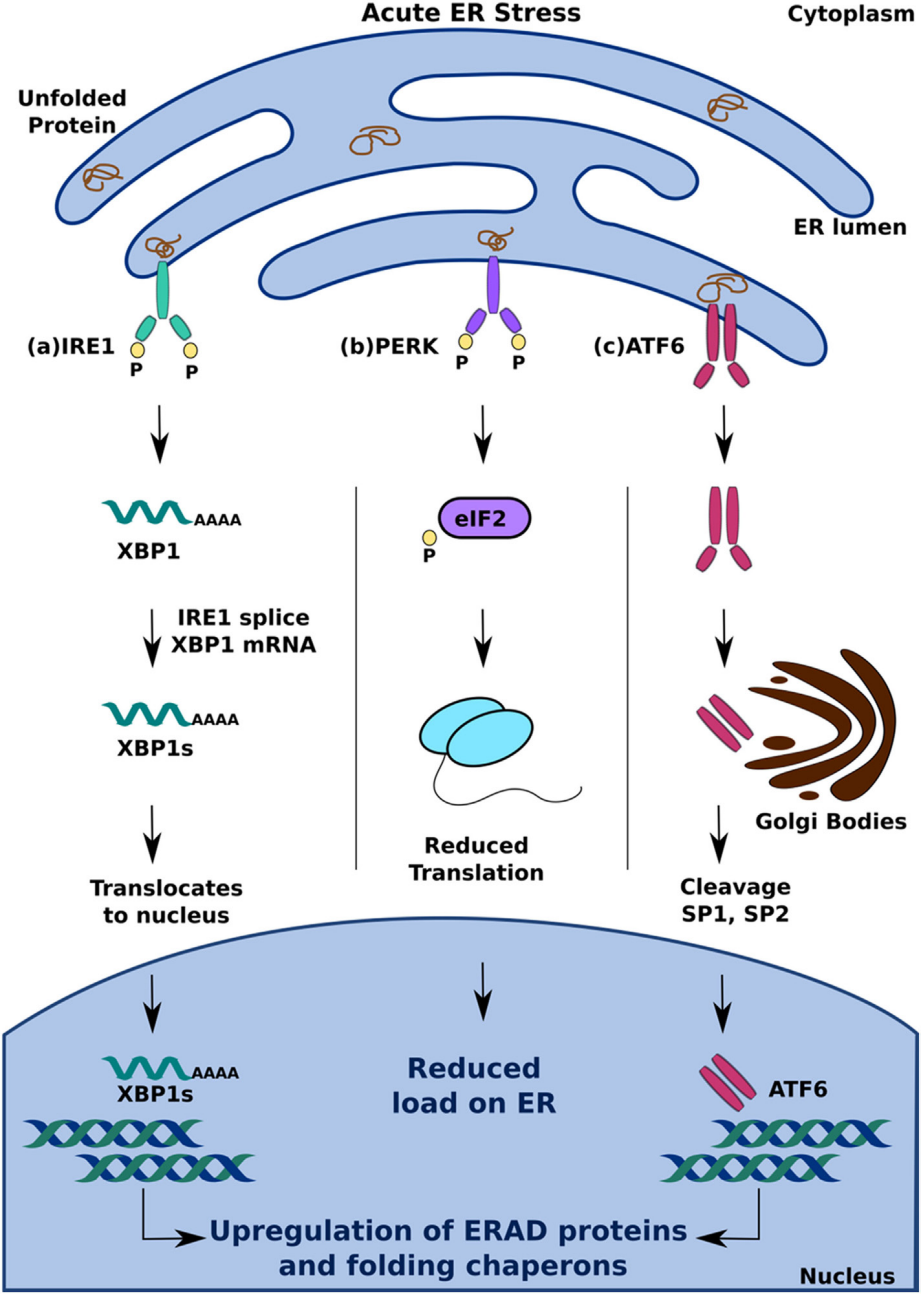
Adaptive unfolded protein response (UPR) signaling under acute endoplasmic reticulum (ER) stress. Accumulation of unfolded protein triggers UPR by activation of **(A)** inositol-requiring 1, **(B)** PERK, and **(C)** activating transcription factor 6. This leads to upregulation of ER-associated degradation protein and folding chaperons to mitigate ER stress and maintain homeostasis.

Inositol-requiring 1 is a type I ER transmembrane kinase that dimerizes and autophosphorylates in the presence of unfolded proteins to become active (27). It also has endoribonuclease activity that, following activation, allows it to splice X-box-binding protein 1 (XBP1) mRNA, which translocates to the nucleus and upregulates the expression of UPR target genes to increase protein folding and degradation (13, 29) (Figure 1A). Another type I transmembrane kinase, PERK, upon activation phosphorylates eukaryotic initiation factor 2 (eIF2) to inhibit protein translation (13, 30). By inhibiting the formation of ribosomal initiation complexes and recognition of start codons, PERK activation results in a decrease in overall translation in the cell, which consequently reduces the load on the ER (27) (Figure 1B). ATF6, a type II ER transmembrane transcription factor, responds to ER stress by translocating to the Golgi, where cleavage by SP1 and SP2 proteases produces an active form of the transcription factor (13, 27). Activated ATF6 can then selectively increase the expression of genes encoding molecular chaperones and ERAD proteins, as well as XBP1, which further amplifies this response (31, 32) (Figure 1C).

**Figure 2:**
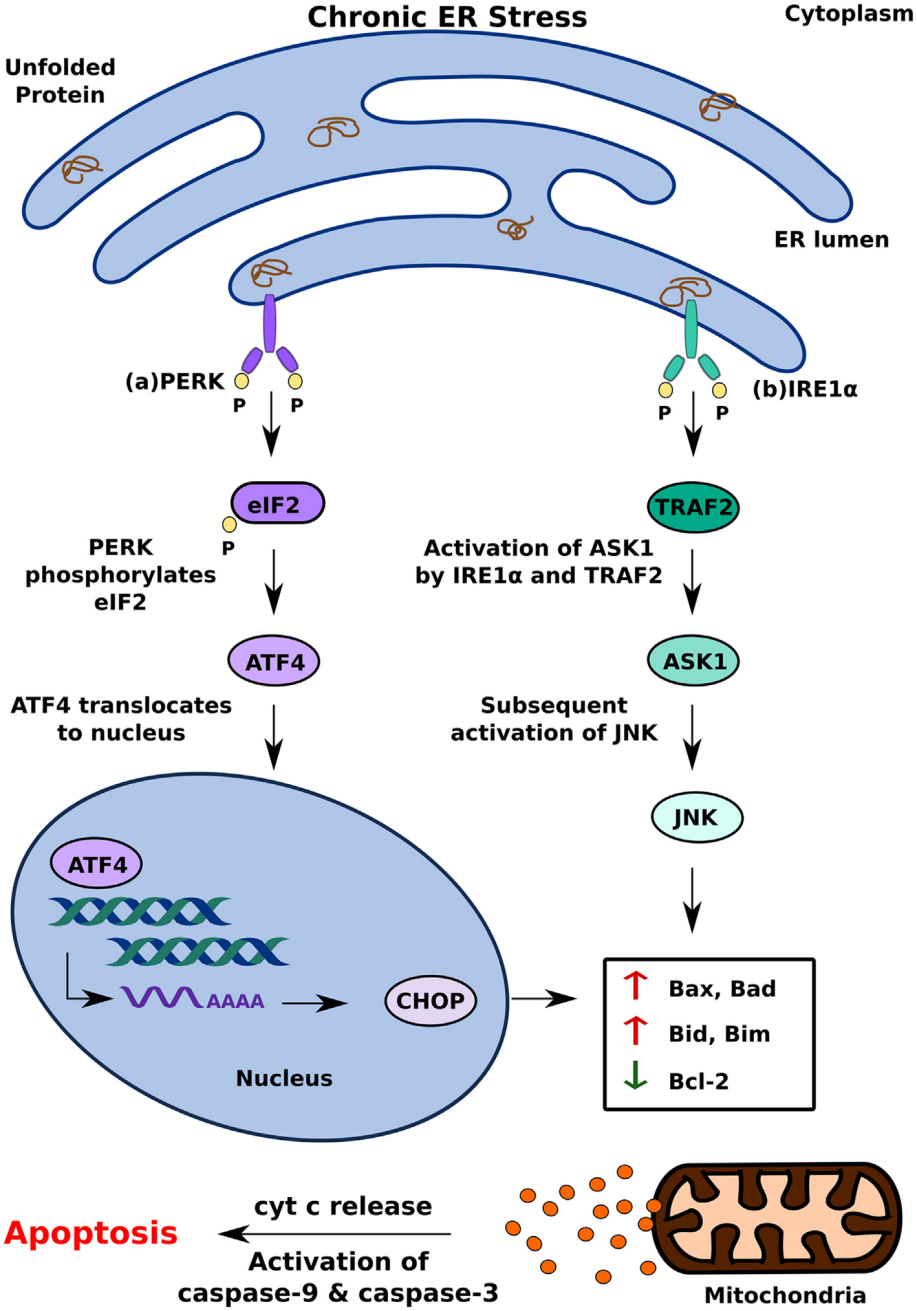
Pro-apoptotic unfolded protein response (UPR) response under chronic endoplasmic reticulum (ER) stress. When UPR is dysfunctional, cell undergoes apoptosis by **(A)** PERK mediated production of CHOP and **(B)** inositol-requiring 1 α-induced activation of ASK1. This leads to cytochrome c (cyt c) release and eventual apoptosis.

Activation of one or more branches of the UPR is typically sufficient to alleviate acute ER stress in a cell; if, however, the UPR is dysfunctional or the cell is exposed to chronic stress, it can cause the cell to undergo apoptosis (27, 33). Of the several ways in which the UPR can induce apoptosis, PERK-mediated production of C/EBP homologous protein (CHOP) is perhaps the best understood. It occurs because of increased eIF2 phosphorylation by PERK, which leads to the activation of the transcription factor ATF4 (16, 29). Translocation of ATF4 to the nucleus increases the expression of genes encoding apoptotic signaling molecules, including CHOP (34). While it is currently unclear how CHOP induces apoptosis in a cell with irreversible ER stress, several studies suggest that altered expression of the principal regulators of apoptotic signaling, Bax and Bcl-2, and upregulation of Death Receptor 5 and Bim contribute to this effect (13, 16, 35) (Figure 2A).

In addition to PERK, IRE1α can induce apoptosis by activating apoptosis signal-regulating kinase 1 (ASK1) through its interaction with TNF receptor-associated factor 2 (TRAF2) (2, 16). Subsequent activation of Jun-terminal kinase (JNK) increases the activity of pro-apoptotic factors Bid and Bax, which initiates the release of cytochrome C into the cytosol through the formation of membrane channels in the mitochondrial membrane (36). By activating two key enzymes of the apoptotic pathway—caspase-9 and caspase-3—cytochrome C triggers a signaling cascade that results in cell death (37). In addition to increasing the activity of pro-apoptotic factors, such as Bid and Bax, JNK also contributes to apoptosis by inhibiting anti-apoptotic factors, such as Bcl-2, which inhibit the release of cytochrome C (36) (Figure 2B).

**Figure 3:**
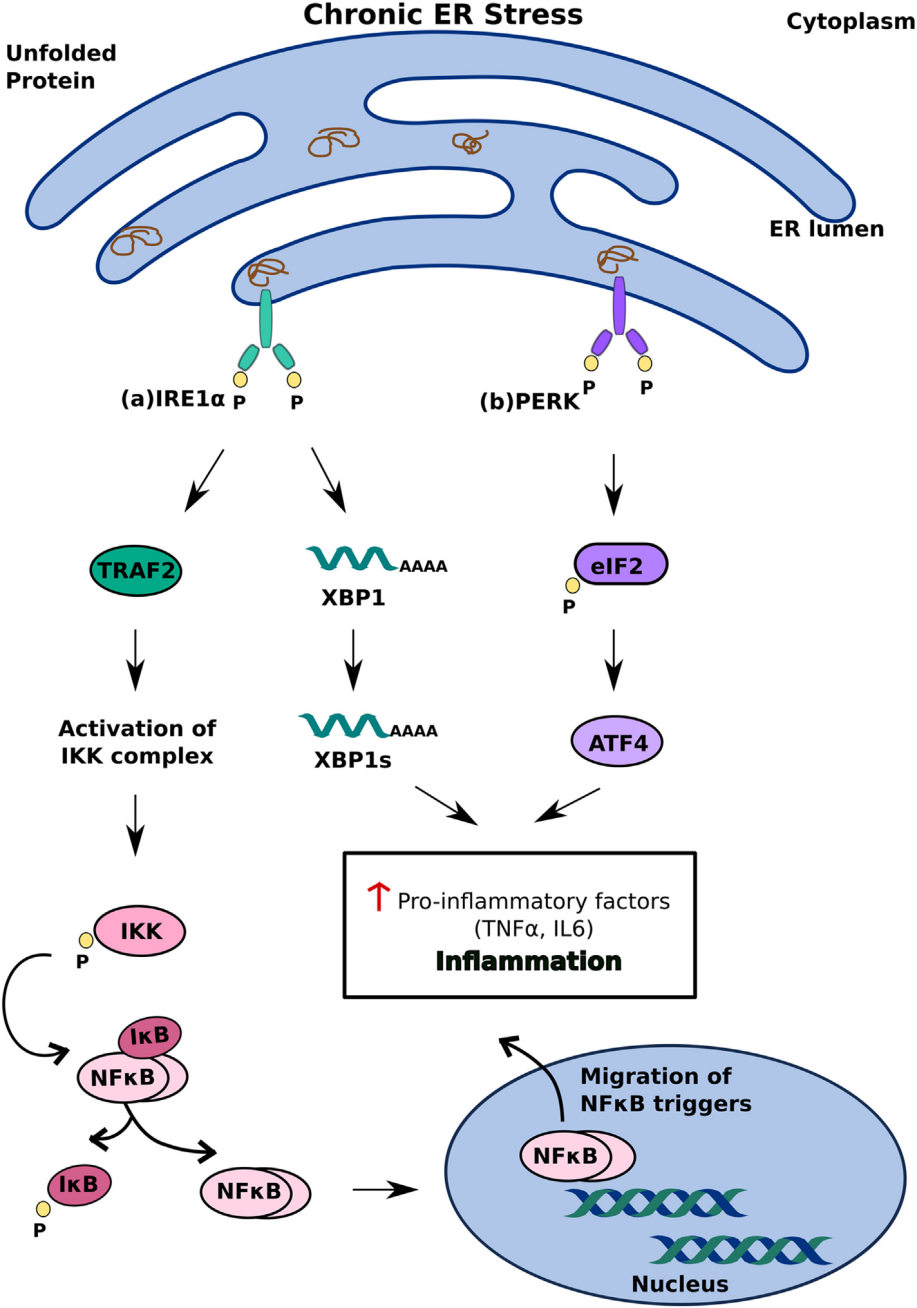
Inflammatory unfolded protein response (UPR) response under chronic endoplasmic reticulum (ER) stress. ER stress triggers apoptosis through activation of inflammation. This can be achieved **(A)** inositol-requiring 1 α-mediated stimulation of IKK complex or XBP1s and **(B)** PERK-mediated activating transcription factor 4 activation. These induce release of pro-inflammatory factors which enhances cell death.

Another model of ER stress-mediated apoptosis proposes that ER stress triggers apoptosis through an inflammatory pathway. The interaction of IRE1α with TRAF2 activates the IKK complex, which can disinhibit NF-κB by inducing the phosphorylation and degradation of Inhibitory κB (38) Migration of NF-κB to the nucleus subsequently triggers pro-inflammatory responses and cell death (39). The decrease in overall translation in the cell secondary to PERK activation further contributes to increased levels of NF-κB as it decreases the translation of IκB mRNA (38) (Figure 3). In addition to NF-κB activation, IRE1α can also trigger inflammation and apoptosis through XBP-1s, which has been shown to increase the production of pro-inflammatory cytokines, such as TNFα and IL6 (40) (Figure 3A). ATF4, which is a product of PERK activation, also contributes to pro-apoptotic signaling by increasing the production of pro-inflammatory factors (40) (Figure 3B). While the precise mechanism by which ER stress induces apoptosis remains to be investigated, it is clear that differential activation of the three key regulators of the UPR—IRE1, PERK, and ATF6—determines the cell’s response to ER stress. Activation of the UPR in response to acute stress facilitates an anti-apoptotic response aimed at alleviating that stress, whereas prolonged activation of the UPR favors pro-apoptotic pathways that produce not only pro-apoptotic factors, such as CHOP and ASK1, but also inflammation (33, 36, 38). Because chronic activation of the UPR can give way to inflammation and cell death, it has now been implicated in inflammatory, autoimmune diseases, including diabetes.

## ER Stress Facilitates the Development of T2DM by Promoting Dysfunction and Apoptosis in β Cells

Pancreatic β cells are unique in their ability to detect changes in blood glucose concentration and respond by altering insulin secretion. To counteract sharp increases in glucose concentration, which occur commonly after a meal, they increase insulin production nearly 25-fold; a response that overloads the ER and compromises its protein folding ability (2). And as the demand for insulin increases, so does the proportion of proinsulin that is misfolded. This misfolded proinsulin then accumulates in the ER and triggers ER stress (12). Under physiological conditions, this ER stress is transient; it occurs due to the sudden increase in blood glucose, and relieves once the glucose levels normalize (27, 41). In diabetics, however, insulin resistance and β cell dysfunction come together to induce chronic ER stress, which eventually results in cell death (2). Several lines of evidence indicate that insulin resistance alone does not, usually, cause type 2 diabetes; most insulin-resistant individuals have various compensatory mechanisms to meet the body’s requirement of insulin, which include an increase in both β cell proliferation and insulin secretion (41–43). In insulin-resistant individuals who do go on to develop T2DM, these compensatory mechanisms are still intact during the early phase of the disease—they lead to an increase, rather than a decrease, in serum insulin levels. As the disease progresses, the increasingly high demand for protein folding makes the β cells particularly susceptible to ER stress, which results in both an increase in misfolded proinsulin and a decrease in insulin production (12, 27). And with progressively more and more proinsulin misfolding and accumulating in the ER, the ER experiences even greater stress, which favors a further reduction in insulin production; this positive feedback loop results in the development of a full-fledged T2DM phenotype.

The most compelling evidence for the role of ER stress in the development of T2DM comes from mutant INS-gene-induced diabetes of youth (MIDY), a syndrome characterized by genetic mutations in the insulin gene that cause it to misfold (12). Although as many as 30 mutations can cause MIDY, one missense mutation, in particular, in *Akita* model mice has been used to study the pathology of the disease. These mice have a C96Y mutation in one copy of *Ins2*, but in spite of two WT *Ins1* alleles and one functional *Ins2* allele, their β cells undergo ER stress-mediated apoptosis secondary to accumulation of misfolded proinsulin in the ER (16). The apoptosis is dependent at least partially on CHOP, as a homozygous disruption in the *Chop* gene rescues the β cells from apoptosis and delays the onset of diabetes by 8–10 weeks (33).

Innumerable studies have documented that Ca^2+^ is one of the key intracellular regulators of insulin secretion and altered Ca^2+^ homeostasis affecting β-cell function and survival (44–47). Previous reports have identified that downregulation of calcium binding protein sorcin (SRI) and associated increased expression of glucose 6-phosphatase C2 in high-fat feeding mice and human islets exposed to palmitate impairs GSIS and reduces glucose tolerance (48, 49). The type 2 ryanodine receptor (RyR2) is yet another Ca^2+^-related protein playing a crucial role in the regulation of insulin secretion and glucose homeostasis. RyR2 forms channel and is involved in transport of Ca^2+^ in multiple cells, including cardiomyocytes and pancreatic β cells. Transgenic mice exhibiting mutations resulting in “leaky” RyR2 channel show intracellular Ca^2+^ leak, activated ER stress response, mitochondrial dysfunction, decreased insulin release, and impaired glucose homeostasis in pancreatic islets and β cells (50). Aberrant glucagon in mice with obese liver activates ER calcium channel, inositol 1,4,5-trisphosphate receptor (IP3R) and results in excessive calcium release into the cytoplasm. This promotes activation of a calcium-sensitive kinase, calcium/calmodulin-dependent protein kinase II (CaMKII). CaMKII activates p38 and MAPKAPK2 (MK2) in hepatocytes initiating CaMKII–p38–MK2 pathway. This mediates increased glucose production by regulating expression of G6pc (glucose-6-phosphatase) and Pck1 (phosphoenolpyruvate carboxykinase) *via* FOXO1 induction. Also, CaMKII represses Atf6 leading to defective insulin signaling (51). Interestingly, corroborative evidence for the severe consequences of ER stress on β cell functioning and survival comes from studies in the mouse model of the Wolfram Syndrome, a rare autosomal recessive disease (16). People with this syndrome have mutations in the *WFS1* gene, which encodes Wolframin, an ER transmembrane protein that modulates Ca^2+^ signaling during ER stress (52). By inhibiting UPR hyperactivity, Wolframin forms an integral part of the feedback loop that maintains glucose homeostasis; mutations in the *WFS1* gene, however, eliminate this response and allow the UPR to continue unchecked (16, 53). Dysregulation of UPR signaling in the Wolfram Syndrome affects multiple cell types and leads to childhood-onset of diabetes mellitus, optic atrophy, and deafness (27). Studies in mice with a heterozygous *WFS1* deletion demonstrate that diabetes develops in those mice as a result of disruption in ER stress signaling (54).

Disruption of even one branch of the UPR can have disastrous consequences on β cell function and survival. The Wolcott–Rallison Syndrome is an autosomal recessive disorder that occurs due to loss-of-function mutations in *EIF2AK3*—the gene that encodes PERK (27). Patients with this syndrome develop diabetes in infancy as well as musculoskeletal impairments, due to multiple epiphyseal dysplasia (16). Without PERK to phosphorylate it, eIF2α is unable to attenuate protein translation in the cell, which leads to a build-up of proinsulin and subsequent ER stress-mediated apoptosis of β cells (30). Several studies in animal models have reported the same phenomenon. Mice with PERK knocked out show all the classic symptoms of Wolcott–Rallison Syndrome, including the onset of diabetes within a few weeks of birth due to progressive loss of β cells (55). A heterozygous mutation in the phosphorylation site of eIF2α also produces a similar phenotype—these mice become obese when given a high fat diet and develop diabetes as a consequence of disrupted PERK signaling (56). Lack of eIF2α phosphorylation, in addition, leads to overproduction of proinsulin and induces ER stress in mice with a homozygous S51A mutation in eIF2α (30). Unlike PERK knockout mice, these mutant mice exhibit β cell dysfunction in late embryonic stages and die shortly after birth due to hypoglycemia. Taken together, these results demonstrate that dysfunction in the PERK-eIF2α signaling pathway disrupts protein homeostasis in β cells and leads to cell death.

## Chronic Hyperglycemia Induces ER Stress in β Cells

Dysregulated translation due to ER stress is at the core of β cell dysfunction and apoptosis in T2DM. It is primarily the product of chronic exposure to high glucose, which facilitates the development of glucotoxicity and insulin resistance, and ultimately leads to the death of β cells (2). While β cells are well-equipped to tackle intermittent increases in blood glucose concentration, chronic high glucose induces long-standing ER stress that activates apoptotic pathways in the cells (57). Corroborative evidence for this phenomenon comes from studies on pancreatic islet sections from patients with T2DM, whose β cells show two classic signs of ER stress pathway activation—an increase in mRNA levels of ER stress markers, including the pro-apoptotic factors, ATF3 and CHOP, and expansion of the ER due to accumulation of proinsulin (58).

Findings from studies using *Akita* mice with a C96Y mutation in *Ins2* also support this model (16, 33). These mice develop progressive hyperglycemia because of a conformational change in insulin that causes it to misfold and accumulate in the ER. Despite an initial increase in the levels of insulin mRNA in homozygous mice at 3 weeks of age, the levels quickly fall off so that by 9 weeks of age, the homozygous mutants have significantly lesser insulin mRNA than control mice. Because these changes mimic fluctuations in the levels of CHOP mRNA, the study attributes the decrease in insulin mRNA to β cell apoptosis (33). The finding that overexpression of mutant *Ins2* in Min6 cells also induces apoptosis lends further support to this hypothesis (33). One model of PERK activation aims to explain the effects of high glucose on β cells, including the initial increase in insulin mRNA levels. It hinges on the antagonistic effects of PERK and protein phosphatase 1 (PP1) on eIF2α and global translation within the cell (59). On one hand, an initial spike in glucose levels after feeding activates PP1, which dephosphorylates eIF2α; the subsequent increase in global translation upregulates the production of insulin to offset the increase in blood glucose concentration (60). On the other hand, chronic exposure to glucose leads to sustained activation of the UPR, which facilitates PERK-mediated phosphorylation of eIF2α (30). Under physiological conditions, the subsequent decrease in global translation serves to alleviate ER stress produced by the accumulation of proinsulin in the ER—a negative feedback loop that ensures translation in the cell remains balanced (61). But, the combination of chronic hyperglycemia and sustained ER stress in T2DM skews this balance in favor of overactivation of the PERK pathway, which leads to excessive phosphorylation of eIF2α. Chronic activity of eIF2α subsequently represses translation of not only insulin but also other key proteins necessary for survival (62). A decrease in the levels of IκB, for example, leads to an increase in the expression of NF-κB; subsequent activation of inflammatory pathways drives apoptosis in the β cell (63). Apoptosis in β cells exposed to salubrinal, an inhibitor of eIF2α dephosphorylation, further supports loss of translational control as the mechanism of glucotoxicity in T2DM (62).

Another model of ER stress-mediated glucotoxicity attributes loss of translational control to errant activity of IRE1α. It proposes that differential activity of IRE1α in response to different concentrations of glucose initiates changes in translation that the β cell cannot tolerate, and in doing so, promotes apoptosis (12, 16, 64). The initial response of IRE1α to high glucose, according to this model, is to act as a positive regulator of insulin production and secretion. Previous studies have shown that IRE1α activation increases insulin secretion in insulinoma cells exposed to high concentrations of glucose, while inhibiting its phosphorylation using siRNAs has the opposite effect (65). The current model suggests a possible explanation—it argues that although it is activated as part of the cell’s physiological response to high glucose, IRE1α, in this context, stays bound to BiP and so does not splice XBP1 mRNA. But if a high concentration of glucose persists, it promotes hyperphosphorylation of IRE1α, which, in trying to restore protein homeostasis, decreases insulin synthesis by cleaving Ins1 and Ins2 transcripts (64). In a typical β cell, this IRE1α activity alleviates ER stress and ensures that insulin production remains proportional to the concentration of glucose.

Because IRE1α’s endoribonuclease action requires it to dissociate from BiP, exposure to high concentrations of glucose increases the levels of both BiP and XBP1. The result of a study with diabetic and nondiabetic patients supports this hypothesis—it found that islets of T2DM patients have higher levels of BiP and XBP1 when exposed to high glucose, as compared to islets of nondiabetics (57). There was, however, no significant difference in the expression of these ER stress markers between the two groups in the presence of low glucose, which confirms that IRE1α activation is specific to a hyperglycemic state (57). This IRE1α-mediated regulation of insulin production in response to changes in glucose concentration is what occurs in a typical β cell. In T2DM, however, chronic activation of IRE1α due to prolonged ER stress leads to excessive cleavage of insulin mRNA and a substantial decrease in insulin production (65). With no insulin to counteract it, glucose levels remain elevated, and initiate a cycle of cellular events that promote ER stress and glucotoxicity. And as the expression of insulin continues to decrease, the β cell, after a point, cannot recover from the translation repression, and undergoes apoptosis. While the precise mechanism of β cell dysfunction and apoptosis remains to be elucidated, it is apparent that loosely regulated translation is the cornerstone of inefficient UPR signaling in T2DM. In addition to directly activating pro-apoptotic pathways, both IRE1α and PERK influence patterns of translation of key proteins in the ER stress and insulin signaling pathways to induce cell death (30, 55, 59, 64). The combination of chronic hyperglycemia, low glucose sensitivity, and insulin resistance in T2DM creates a cellular environment that exacerbates this response, and ultimately leads to glucotoxicity in the β cells.

## Changes in Patterns of miRNA Expression are Linked to the Development and Progression of T2DM

The development of a disease phenotype in T2DM does not depend solely on dysregulation of translation of key proteins—it is subject to changes in transcription that favor glucose intolerance and impair insulin secretion. Over the past few years, miRNAs have emerged as the focus of several studies investigating the genetic basis of T2DM (66–68). Perhaps, the strongest evidence of importance of miRNAs in the regulation β cell function comes from a study on mice that have a β cell-specific disruption of Dicer1 (69). Owing to the lack of Dicer1, an endoribonuclease necessary for miRNA biosynthesis, these mice are unable to correctly process and generate β cell-specific miRNAs. The result is decreased insulin gene expression and impaired insulin secretion. Over time, the disruption in insulin signaling gives way to chronic hyperglycemia and, eventually, full-blown T2DM. Dicer1 mutant mice also display morphological changes—they have altered islet architecture and reduced β cell mass—that correspond with the development of the disease phenotype (69). These results demonstrate that impaired miRNA processing can have a profound impact on the development of T2DM. The opposite also holds true—diabetics show a tissue-specific reduction in the expression of Dicer1, which supports the argument that miRNA processing and development of disease pathology is a two-way street (70).

Following these studies on Dicer1, investigators have been able to pinpoint specific miRNAs that are differentially expressed in T2DM patients as compared to healthy non-diabetics. Of the many miRNAs that have been found to regulate β cell function, miR-375 is one of the first, and probably the best, characterized. On one hand, it is found to be upregulated in pancreatic tissue from T2DM patients, where its overexpression leads to a decrease in GSIS, β cell mass, and β cell proliferation (71). Profiling of primary human islets and enriched β cell preparations has also established that this miRNA is overexpressed in these tissues (66). On the other hand, a miR-375 knockout produces hyperglycemia in mice and increases both glucose biosynthesis and blood glucose concentration (72). Leptin-deficient *ob/ob* mice—the mouse model for obesity-related diabetes—with miR-375 knocked out also experience a reduction in β cell mass that leads to diabetes (72). While further investigation is necessary to establish the role of miR-375 in regulation of β cell function, these results, taken together, suggest that dysregulation of miR-375 contributes to the development of diabetes. Like miR-375, a study found that miR-7 is also highly overexpressed in pancreatic islets as compared to the adrenal glands (73). When overexpressed in decompensating BKS *db/db* mice—these mice are leptin receptor-deficient and cannot increase insulin secretion to compensate for insulin resistance (74)—the increased expression of miR-7a leads to chronic hyperglycemia and impaired insulin secretion. miR-7 overexpression, however, does not affect β cell proliferation and apoptosis. Results of the same study with mice lacking miR-7a2, a precursor for miR-7, also suggest a role for this miRNA in regulating glucose homeostasis (73). By regulating genes in the insulin secretory pathway—specifically those coordinating fusion of insulin granules with the plasma membrane—the miR-7a2 knockout improves glucose tolerance by increasing insulin secretion (73). The overall effect of decreased miR-7 on the diabetic phenotype is still unclear, but its positive effect on glucose tolerance and insulin secretion makes it a valuable therapeutic option to treat T2DM.

Several lines of evidence also converge on miR-124 as a modulator of insulin secretion and glucose metabolism. Not only is it more highly expressed in islets of T2DM patients in comparison to healthy donors but its overexpression in Min6 cells also impairs GSIS significantly when the glucose concentration is high (75). Silencing miR-124, conversely, promotes the expression of genes that regulate β cell function including *Mtpn*, the gene encoding myotrophin, *NeuroD1*, a transcription factor that regulates insulin gene expression, and *Akt3*, a kinase in the anti-apoptotic pathway (75). Another target of miR-124 is FoxA2, a transcription factor that mediates pancreatic development, β cell differentiation, glucose metabolism and insulin secretion (76). The levels of FoxA2 correlate inversely with those of miR-124—when miR-124 is overexpressed, it leads to decreased expression of FoxA2 and its downstream targets, which impairs both insulin biosynthesis and secretion. The effects of miR-124 overexpression on insulin secretion are not, however, clear cut. Greater expression of this miRNA typically leads to an increase in the expression of *Snap25, synapsin-1*, and *Rab3A*—all of which are genes encoding proteins in the exocytotic pathway—while reducing the expression of *Rab27A* and the gene encoding its effector protein, *Noc2* (17). Because miR-124 can upregulate or downregulate different components of the exocytotic pathway independently, it is difficult to predict its overall effect on insulin secretion. One model of miRNA-mediated regulation of insulin secretion proposes that the net effect depends on the metabolic context (17). When glucose levels are low, higher expression of miR-124 in Min6 cells causes an increase in insulin secretion; this trend, however, reverses when the glucose levels get too high. Even though miR-124-mediated regulation of insulin function is not clearly understood, these results implicate miR-124 as a key modulator of insulin signaling pathways involved in T2DM.

Apart from miR-375, miR-7, and miR-124, numerous other miRNAs have been reported to be upregulated in islets of T2DM patients as compared to their non-diabetic counterparts (21, 66, 77, 78). A recent study, however, has identified miR-155, a miRNA that is present in lower concentrations in the serum of T2DM patients (79). Overexpression of this miRNA in transgenic RL-m155 mice correlates with lower levels of blood glucose in both the fasting and the fed states and allows these mice to clear glucose more efficiently than their control littermates. In addition to glucose clearance, RL-m155 mice also have better insulin sensitivity, owing to better sensitivity in peripheral tissues such as liver, adipose tissue, and skeletal muscle; the same effect extends to RL-m155 mice kept on a high-fat diet.

A homeostatic model of assessment of insulin resistance (HOMA-IR) supports the finding that higher expression of miR-155 improves insulin sensitivity (79). The HOMA-IR index is a measure of insulin sensitivity—it has low values indicating optimal insulin sensitivity and high values corresponding to different degrees of insulin resistance (80). In serum samples from T2DM patients, it correlates negatively with the levels of miR-155, which strengthens the argument that higher levels of miR-155 contribute to better insulin sensitivity (79). This correlation also implies the opposite—that the lower levels of miR-155 seen in serum samples from T2DM patients are linked to poor insulin resistance in those individuals. But whether downregulation of miR-155 in diabetics is a cause of insulin resistance or a product of it requires further investigation.

Even though the specific mechanisms by which miRNAs regulate glucose metabolism, especially in the context of T2DM, are currently unclear, studies exploring miRNA profiles of patients and healthy individuals have been instrumental in characterizing the pathways involved. A profiling study by Bunt et al. (66) has identified 366 miRNAs that are expressed in human islets, of which 346 are expressed specifically in β cells. Comparison of miRNA expression in islets versus other tissues, such as skeletal muscle, liver, and adipose tissue, revealed that 40 of the 366 islet miRNAs are expressed exclusively in islets. And of these 40 miRNAs, there are some, such as miR-375, that are known to influence insulin biosynthesis and secretion, while others constitute novel finds whose role in T2DM pathogenesis needs further investigation.

To explore how some of these miRNAs contribute to diabetes pathogenesis, the investigators next analyzed genome-wide association data from T2DM patients and non-diabetic individuals (66). By studying the predicted target sites of islet miRNAs, they identified 6496 gene variants that overlap with variants known to associate with T2DM. One of these variants is in the 3′ UTR of the SLC30A8 locus, which encodes a zinc transporter protein involved in insulin secretion (81), and shows genome-wide association for T2DM. A similar correlation emerged when the investigators searched for variants that overlap with miRNA target sites within 58 loci known to be associated with T2DM—they found 10 variants that map to 6 loci, all of which are linked to T2DM pathogenesis (66). This finding that the relationship between miRNA target sites and T2DM-associated variants is bidirectional makes for a strong case that miRNAs have a contributory role to play in T2DM pathogenesis.

Findings of several other profiling studies support the hypothesis that differential expression of miRNAs influences insulin signaling and glucose metabolism. Bolmeson et al.’s (82) study of miRNA expression patterns uncovered 319 miRNAs that are expressed in islets from healthy donors, of which 5 have significantly higher expression in islets than liver or skeletal muscle. The expression of three of these five miRNAs—miR-184, miR-375, and miR-127-3p—correlates positively with insulin gene expression. Because previous studies have implicated miR-375 in T2DM pathogenesis (66, 72, 83), it is plausible that downregulation of the other two miRNAs also contributes to the diabetic phenotype. But until investigators can ascertain the role of these miRNAs in T2DM, the search for potential biomarkers can benefit from studies exploring miRNA expression in islets from diabetic donors, instead of healthy donors alone. miRNA profiles of islets from prediabetic and diabetic mice suggest that the levels of certain miRNAs—miR-132, miR-184, and miR-388-3p—begin to change even before the onset of the disease (71). These initial changes have a positive effect on β cell function—they increase both the mass and activity of β cells and promote insulin secretion—but dysregulation of other miRNAs after onset of the disease favors apoptosis of those cells and worsening of the diabetic phenotype. miR-27a-3p and miRNA-29 family members are possible regulators of peripheral insulin sensitivity and are found to be upregulated in skeletal muscles of human type 2 diabetic patients than normal obese/overweight individuals (84). Plasma levels of miR-122, miR-99, let-7d, miR-18a, miR-18b, miR-23a, miR-27a, miR-28, and miR-30d show differential expression between non-progressors with impaired glucose tolerance and subjects with normal glucose tolerance or T2DM (85). let-7a and let-7f involved in the regulation of the adiponectin pathway are significantly decreased in treatment-naive diabetic patients and antidiabetic treatment reversed this trend (86). System review approach has identified miR-148b, miR-223, miR-130a, miR-19a, miR-26b, and miR-27b as potential circulating biomarkers and miR-146a and miR-21 as potential tissue biomarkers in T2DM (87). However, such unique microRNA profile can aid in predicting diabetic development provided these results are replicated using larger cohorts. miRNAs involved in associated complications of diabetes like, diabetic neuropathy, nephropathy, retinopathy, and cardiovascular complications have also been identified (88, 89). Patients with diabetes are predisposed to development of diabetic foot ulcers (DFU). However, mRNA and miRNA profiling between diabetic and healthy foot skin do not uncover any major significant change in the two groups. Although it is suggested that additional factors, such as neuropathy, vascular complications, or duration of DM, may changes foot skin biology making it susceptible for healing impairment and development of DFU (90). Additional miRNAs implicated in T2DM and its associated complication is listed in Table 1.

**Table 1:** miRNAs implicated in T2DM and associated complications.

miRNA	Status	#Potential mRNA targets	Reference, UID
**(a) Expression profile in peripheral blood/serum/plasma**			
miR-375, miR-9	Upregulated	–	(92), 29373500
miR-34a, miR-125b	Upregulated	–	(93), 29285097
miR-7	Upregulated	–	(94), 28646700

**(b) Pancreatic (cell dysfunction)**			
miR-26a	Downregulated	PTEN	(95), 29191656
miR-199a-5p	–	SIRT1	(96), 28338182
miR-130a, miR130b, miR-152	Upregulated	PDHA1, GCK	(97) 28332581
miR-463-3p	Upregulated	ABCG4	(98), 27664094

**(c) Hepatic insulin sensitivity**			
miR199a-5p	Upregulated	ATG14	(99), 29540751
miR-222	Upregulated	IRS1	(100), 29364977
miR-125b	Upregulated	PIK3CD	(101), 29319168
miR-21	Downregulated	FOXO1	(102), 28627440
miR-338-3p	Downregulated	PP4R1	(103), 28467989

**(d) Diabetic neuropathy, nephropathy, retinopathy**			
miR-146a	Downregulated	IL-1β, TNF-α, NF-κB	(104), 29398906
miR-199a-3p	Upregulated	SerpinE2	(105), 28677735
miR-133b, miR-342, miR-30a	Upregulated	–	(106), 27470555
miR-503, miR-181d	Upregulated	–	(107), 27770539
miR-93	Upregulated	–	(108), 28382439

**(e) Cardiovascular complications and obesity**			
miR-126, miR-132	Downregulated	–	(109), 28065883
miR-29	Upregulated	Lypla1	(110), 29374012
miR-130b	Upregulated	PPARγ	(111), 27746169
miR-31	Upregulated	GLUT4, PPARγ, IRS1, ACACA	(112), 29605715

*#Potential mRNA targets*.

*ACACA, acetyl-CoA carboxylase alpha; ABCG4, ATP-binding cassette A4; ATG14, autophagy related 14; FAM3A, family with sequence similarity 3 member A; FOXO1, forkhead box O1; GLUT4, glucose transporter type 4; GCK, glucokinase; IRS1, insulin receptor substrate 1; IL-1β, interleukin 1 beta; Lypla1, lysophospholipase I; NF-κB, nuclear factor kappa B subunit 1; PPARγ, peroxisome proliferator activated receptor gamma; PTEN, phosphatase and tensin homolog; PIK3CD, phosphatidylinositol-4,5-bisphosphate 3-kinase catalytic subunit delta; PP4R1, protein phosphatase 4 regulatory subunit 1; PDHA1, pyruvate dehydrogenase E1 alpha; SerpinE2, serpin family E member 2; SIRT1, sirtuin 1; TNF-α, Tumor necrosis factor α*.

Multiple studies have demonstrated miRNAs as possible biomarkers but studies identifying therapeutic miRNA targets are limited. For instance, anti miRNA oligonucleotide (AMOs) for miRNAs (anti-miR-181a, anti-miR-320, etc.) adequately revert miRNA to normal levels and correct defected insulin signaling (91). However, the use of AMOs is not recommended due to resistance in its uptake by the intended tissues. This indicates that more studies are required to explore alternate ways by which miRNAs can be targeted and modulated for better management of T2DM.

## Discussion and Future Perspectives

Type 2 diabetes mellitus evolves from a complex interplay between genetic and environmental factors, but defects in ER stress signaling are evidently crucial for the development of glucose intolerance and insulin resistance. Activation of the UPR is a key step in managing the effects of glucose on β cells—its adaptive, anti-apoptotic responses allow these cells to tolerate acute changes in glucose concentrations, but chronic exposure to high glucose tips the scales in favor of maladaptive, pro-apoptotic activity that promotes β cell dysfunction and cell death (27, 64). What continues to evade investigators is which molecular switches mediate the transition between these two phases of the UPR, and how this transition triggers apoptosis. Both ATF4 and ATF6 can regulate apoptosis in the cell, but findings of several studies currently regard the induction of CHOP as the initiator of pro-apoptotic activity (33, 113–115). The severe effect of a PERK knockout on islet morphology and function clearly indicates that intervention that far upstream has disastrous consequences for the cell as it affects cellular processes that are essential for survival (30). Decreasing the expression of CHOP, however, inhibits apoptosis and preserves β cell function (33, 116, 117). This finding, more importantly, suggests a therapeutic role for inhibitors of CHOP induction in the treatment of T2DM.

In addition to drugs that directly inhibit CHOP, another class of drugs that have shown therapeutic potential is inhibitors of glycogen synthase kinase 3 (GSK3). UPR activation is only aspect of ER stress signaling, and several other pathways work in parallel to influence the cell’s response to stress. One such pathway is the PI3K/Akt pathway, which, under physiological conditions, prevents apoptosis by inhibiting GSK3 and its downstream, pro-apoptotic target, Caspase-3 (118). Evidence of crosstalk between the PI3K/Akt pathway and ER stress signaling suggests that activation of GSK3 can also induce apoptosis by increasing the expression of CHOP (119). GSK3 inhibitors, conversely, decrease CHOP expression and protect several types of neuronal cells from drug-induced ER stress (118). By developing inhibitors that mimic the effects of PI3K/Akt activation and recapitulate this effect *in vivo*, investigators may be able to reduce the extent of β cell death in T2DM.

**Figure 4:**
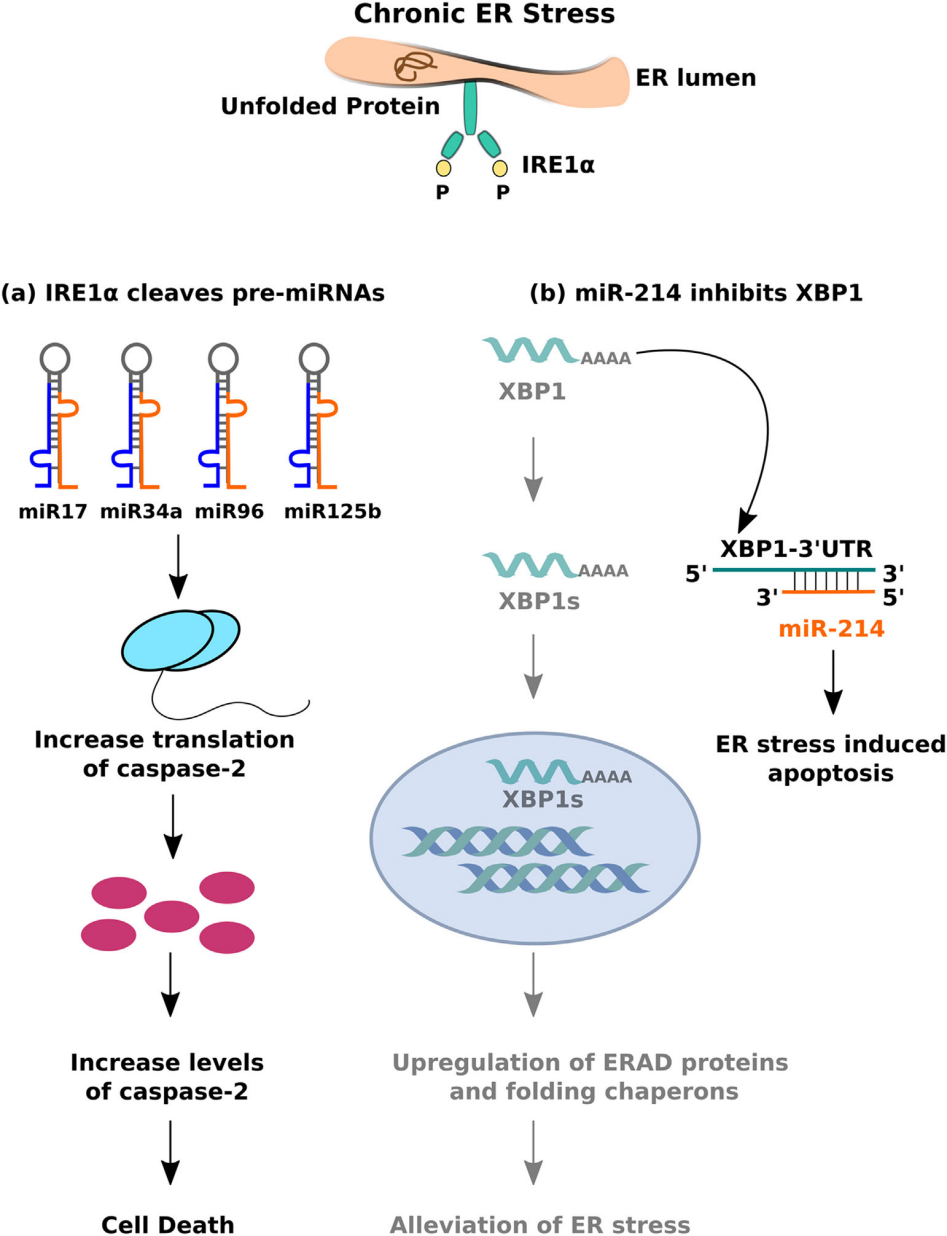
Crosstalk between miRNA and inositol-requiring 1 α signalling. **(A)** The endoribonuclease activity of IRE1α cleaves set of pre-miRNAs (miR-17, miR-34a, miR-96, and miR0125b) leading to decreased expression of mature miRNAs, thereby resulting in caspase-2 upregulation and eventual cell death. **(B)** miR-214 binds to 3′-UTR (untranslated region) of XBP-1, leading to mRNA downregulation, and inhibition of IRE1α mediated adaptive unfolded protein response (UPR) response.

Another model of ER stress-mediated apoptosis proposes that hyperactivity of the IRE1α branch of the UPR can induce apoptosis by regulating the levels of specific miRNAs. Prolonged ER stress leads to sustained activation of IRE1α, whose intrinsic endoribonuclease activity allows it to cleave a set of 4 miRNAs—miR-17, miR-34a, miR-96, and miR-125b—which typically inhibit the translation of caspase-2 (120). The result is an increase in the levels of caspase-2 and subsequent cell death (Figure 4A). What makes this finding particularly important is that it indicates crosstalk between the miRNAs and ER stress signaling pathways. Several lines of evidence support the hypothesis that bidirectional interactions occur between these pathways and influence disease phenotypes. Studies on HCC demonstrate that greater activity of the UPR correlates with lower levels of miR-122, which leads to reduced apoptosis in human hepatoma cells (121). The same miRNA, when overexpressed in those cells, inhibits UPR activation and thereby makes them susceptible to the effects of anti-cancer drugs. miR-214 shows a similar effect—it typically inhibits XBP-1 and makes hepatoma cells susceptible to ER stress-induced apoptosis; XBP-1 activity, in turn, reverses the effects of miR-214 by reducing its expression (122) (Figure 4B). In human cardiomyocytes, ER stress results in the downregulation of miR-702, which promotes apoptosis, but transfection of miR-702 into those cells attenuates this response (123). Taken together, these results strongly indicate that bidirectional interactions between miRNAs and ER stress signaling pathways may regulate apoptosis of β cells in T2DM as well.

These interactions are particularly important in the context of diagnosis of T2DM—they suggest that changes in miRNA expression can be an early marker of chronic ER stress in β cells and, by extension, β cell dysfunction and apoptosis. Already there is evidence that the levels of some miRNAs begin to fluctuate even before the onset of the disease, and that these fluctuations can be easily detected in the sera of those patients (17). Both miR-23 and miR-126, for example, are significantly downregulated in the sera of type 2 diabetics in comparison to prediabetic and nondiabetic individuals (77, 78). But although miRNAs show promise as potential biomarkers of T2DM, one key question remains to be answered—Does a change in the expression of specific miRNAs cause defects in insulin signaling or is it a result of those defects? Many studies have drawn a correlation between the levels of miRNAs in healthy and diabetic individuals, and deficits in insulin secretion and glucose tolerance, but how those miRNAs influence T2DM pathogenesis is not clear-cut. Investigators have found it difficult to ascertain the precise relationship as miRNAs target many genes and often regulate the expression of those genes in a tissue-specific manner. Some changes in miRNA expression also do not correlate necessarily with a worsening of disease pathology. Expression of miR-132, miR-184, and miR-338-3p, for example, begins to change before the onset of T2DM and, in fact, improves β cell mass and function (71). Because it has a positive effect on β cells, this change in miRNA expression could be part of an underlying physiological mechanism to combat early glucose intolerance. A greater understanding of the extent to which changes in miRNA expression reflect the progression of disease pathology will likely aid in narrowing down potential biomarkers to detect T2DM early.

## Conclusion

Despite the inherent complexity of T2DM, miRNAs, and ER stress have emerged as key regulators of transcriptional and translational responses that control insulin signaling. Studies were done in animal models of T2DM and obesity strongly indicate a role for chronic ER stress and dysregulated UPR signaling in the pathogenesis of T2DM. Normalizing the activity of the UPR is arguably one of the most important goals of T2DM therapy, and further insight into the mechanisms of ER stress-mediated apoptosis will likely aid in the development of drugs that can restore protein homeostasis and prevent the death of β cells. Increasing prevalence of T2DM, however, warrants not only better treatment, but also early diagnosis to manage symptoms earlier and prevent the development of related pathologies. miRNAs are particularly promising in this regard, as changes in their expression frequently correlate with the spread of disease pathology and various aspects of β cell function. A superficial understanding of how those miRNAs regulate gene expression to influence the T2DM phenotype has, so far, limited their use as biomarkers. But with future investigations offering deeper insight into the specifics of miRNA regulation of gene expression, miRNAs have the capacity to revolutionize the way investigators diagnose and treat T2DM.

## Author Contributions

BB and CB conceived of the presented idea and wrote the review. ML contributed in writing of the final version and designed the figures.

## Conflict of Interest Statement

The authors declare that the research was conducted in the absence of any commercial or financial relationships that could be construed as a potential conflict of interest.
